# Technology and Heart Failure Therapeutics (THT2022): A Glimpse Into the Future of Devices and Novel Therapies for Heart Failure

**DOI:** 10.1016/j.jacbts.2022.02.011

**Published:** 2022-04-04

**Authors:** Daniel Burkhoff, Juan F. Granada, Martin B. Leon, the Cardiovascular Research Foundation and the Co-Directors of Technologies and Heart Failure Therapeutics

**Affiliations:** a Cardiovascular Research Foundation, New York, New York, USA; b Division of Cardiology, Columbia University, New York, New York, USA

**Figure:**
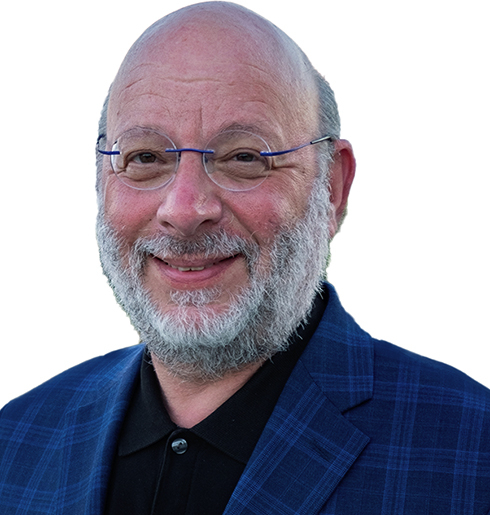

For decades, the cornerstone of heart failure (HF) treatment has focused on medical therapies, such as diuretics, afterload reducing agents, beta-blockers, and mineralocorticoid antagonists. During the 1990s, research showed the beneficial effects of these agents on hemodynamics, exercise tolerance (peak Vo_2_ and 6-minute walk test), HF exacerbations, and mortality. The late 1990s saw the dawn of device-based treatments for HF when left ventricular assist devices were first approved by the US Food and Drug Administration as a bridge to transplant. This was followed in the early 2000s by US Food and Drug Administration approval of cardiac resynchronization therapy, where clinical benefits similar to those observed with drugs were seen in patients with medically refractory HF. Implantable cardioverter-defibrillators were also introduced for primary prevention of sudden cardiac death. The success of these device-based therapies, which are indicated to treat relatively small and specific subsets of patients with HF, inspired a generation of innovators to develop new devices to address the myriad of unmet needs encountered by patients with HF, as well as the health care systems tasked with providing their short- and long-term care.

Part of the enthusiasm that motivated device development was the perception that devices could be brought to market more efficiently than drugs, wherein the development pathways can be markedly protracted. Perhaps unexpectedly, the past 2 decades have brought several important new drugs to market and adoption, but only a few devices survived the approval gauntlet. Nevertheless, whereas drugs have reduced mortality and HF hospitalizations, significant residual risk for these events persists. Progression of HF has been inexorable despite optimal medical therapy, and quality of life remains poor for many patients with HF. Thus, the quest to develop new adjunctive device-based therapies for HF remains.

The Cardiovascular Research Foundation has been at the forefront of showcasing technologies for coronary and structural heart diseases since 1988 at its flagship Transcatheter Cardiovascular Therapeutics (TCT) and Transcatheter Valve Therapies (TVT) meetings. In the same spirit, the inaugural Technologies and Heart Failure Therapeutics meeting,^1^ held on February 1 and 2, 2022, in New York City, was born out of the desire to integrate the efforts of clinicians, innovators, regulators, and thought leaders who are tackling the epidemic of HF from many and dramatically different approaches. More than 500 in-person and 2,000 online participants reviewed the latest in drug and device-based therapies and discussed what the future holds for diagnostics, management strategies, and therapies for HF.

One of the highlights of Technologies for Heart Failure Therapeutics meeting was the “Shark-Tank” innovation competition, where 7 start-up companies presented their concepts to an esteemed panel of clinicians, regulatory experts, and investors. Company representatives summarized the fundamentals of their technology including intellectual property position, available preclinical and clinical data, regulatory strategy, and their ultimate business model, in hopes of capturing the vote of the judges as the company most likely to succeed. Technologies ranged from monitoring devices with machine learning backends to devices for acute decompensated and chronic HF. This special edition of *JACC: Basic to Translational Science* includes Brief Reports summarizing the presentations delivered by all 7 companies along with video links to their presentations and the discussion by the judges. Collectively, these reports highlight the process of translational research and serve to inspire readers to join the quest to develop new devices for the treatment of HF!

## Funding Support and Author Disclosures

The authors have reported that they have no relationships relevant to the contents of this paper to disclose.
